# Time Spent on Private Tutoring and Sleep Patterns of Chinese Adolescents: Evidence from a National Panel Survey

**DOI:** 10.3390/children10071231

**Published:** 2023-07-17

**Authors:** Yueyun Zhang

**Affiliations:** Department of Sociology, School of Humanities, Social Sciences & Law, Harbin Institute of Technology, Harbin 150001, China; zyueyun@hit.edu.cn

**Keywords:** private tutoring, sleep, adolescents, China

## Abstract

Objective: In recent decades, there has been a marked increase in private tutoring and a decline in sleep health among adolescents. This study aimed to outline the association between time spent on private tutoring and sleep patterns of Chinese adolescents. Methods: Participants were from a nationwide two-wave panel survey. We performed OLS regressions of sleep duration at wave 2 and Poisson regressions of sleep problems at wave 2 on tutoring time at wave 2, adjusting for baseline sleep duration and other covariates. Tutoring time was assessed by three items: daily hours of tutoring, daily hours of tutoring on weekdays, and daily hours of tutoring on weekends. Results: Overall, more time spent on private tutoring was associated with shorter sleep duration and more sleep problems. Furthermore, both weekday and weekend tutoring can have a positive effect on the number of sleep problems. With regard to sleep duration, weekend tutoring time played a negative role, while the effect of weekday tutoring time was negligible. Conclusion: In the Chinese context, which is characterized by intense academic competition, participation in private tutoring plays a significant role in shaping students’ sleep duration and sleep problems. To improve the sleep health of adolescents, the time spent on private tutoring should be carefully monitored and regulated.

## 1. Introduction

Sleep is one of the most important health behaviors for everyone. It plays a critical role in maintaining health and well-being in all age groups [1]. Especially for adolescents, who are experiencing pronounced physiological and psychological changes, adequate sleep can significantly contribute to better emotional regulation and higher quality of life [2]. However, inadequate sleep quantity or quality is a common problem for adolescents [3] due to both biological and sociocultural reasons [4,5]. Furthermore, time-trend observations have documented a worrying decline in adolescent sleep duration in many societies over the past few decades [6,7,8,9].

Because a significant proportion of adolescents are still in school, academic activities centered around schoolwork have often been identified in previous literature as potential risk factors for adolescent sleep health. Increased time spent on homework has been associated with decreased nighttime sleep [10,11] and increased difficulty in maintaining sleep throughout the night [12]. The academic stress of longer school hours may also disrupt sleep. For example, a study using data from 915,054 adolescents from 36 European and North American regions reported an overall increase in perceived school pressure between 2002 and 2018, and school pressure was associated with more frequent health complaints, such as difficulty falling asleep [13]. Indeed, there is growing evidence that the recent increase in health complaints among adolescents may be closely related to the intensive use of social media in Western contexts [14], whereas in Asian societies, such as South Korea and China, the burden and stress associated with academic activities may act as a more important risk factor for adolescent health than social media use [9].

This study paid particular attention to the role of time spent on private tutoring in shaping the sleep patterns of adolescents in China. Private tutoring is an umbrella term that encompasses a variety of out-of-school learning practices designed to help students improve their educational performance [15]. Also known as shadow education, it imitates the formal education sector by providing similar courses and learning materials [16,17]. East Asian societies such as Japan and South Korea have long embraced private tutoring [18,19], while it is now commonplace in Western societies as well [20,21]. In fact, data from the Program for International Student Assessment (PISA) show an upward trend in tutoring practices worldwide from 2003 to 2012 [17]. In 2012, more than 30 percent of 15-year-old students in Germany, Hong Kong, Latvia, Russia, Spain, Turkey, and Uruguay spent some time each week in private tutoring, while nearly half of students in Brazil, Greece, South Korea, and Thailand did so [17].

Two perspectives that have emerged in prior literature are helpful in understanding the relationship between time spent on private tutoring and adolescents’ sleep patterns. The “displacement” perspective views private tutoring activities as concrete behaviors that are time-consuming and thus likely to displace other extracurricular activities (e.g., sports or recreation) that may be beneficial to sleep quality, or to displace some nighttime that would otherwise be devoted to sleep [22,23]. The “stress” perspective views private tutoring as a continuation and extension of learning practices, and thus similar in nature to homework [24]. Since academic stress often arises from the intense workload of homework, academic stress may also arise from the extended hours of private tutoring. Both perspectives suggest that greater involvement in tutoring leads to poorer sleep quality.

To our knowledge, only a handful of studies have examined the relationship between private tutoring and adolescent sleep patterns, mostly in East Asian societies. In Singapore and South Korea, more time spent on homework and private tutoring was found to be related to shorter sleep durations [9,11,25]. In Hong Kong, ethnographic studies have speculated that tutoring may disrupt students’ daily routines, forcing them to delay eating, bathing, and sleeping [26]. In mainland China, a questionnaire survey of 2149 students in 16 elementary schools found that private tutoring significantly reduced students’ sleep time [27]. Another study of middle school students found that more tutoring time was associated with poorer sleep quality [24].

This study aimed to extend previous research in three important ways. First, we focused on both the quantity and quality of sleep in understanding adolescent sleep patterns. Both hours of sleep per night and number of sleep problems were examined. Second, in measuring time spent on private tutoring, we additionally distinguished between weekday tutoring time and weekend tutoring time, adding elaborations on the associations between private tutoring and sleep patterns. Third, we used data from a national panel survey so that our findings can be better interpreted in a causal framework and generalized with greater confidence to the target population of Chinese adolescents. Our findings have policy implications for the development of tailored interventions to improve adolescent sleep health in Asian societies, as well as in other regions experiencing the expansion of private tutoring and the deterioration of child and adolescent sleep.

## 2. Participants and Methods

### 2.1. Participants and Data Collection

Participants were drawn from a two-wave panel survey that is part of a nationally representative panel survey of Chinese middle school students, the China Education Panel Survey (CEPS). The CEPS is administered by the National Survey Research Center at the Renmin University of China. It is an ongoing longitudinal survey project that focuses on various aspects of the lives of Chinese in-school adolescents, including academic activities, physical health, and socioemotional well-being. The baseline survey, which was conducted during the 2013–2014 school year, used a stratified, multistage probability proportional to size sampling design. The sample selection process involved dividing the country into three strata based on the 2010 national census, followed by the selection of 28 county-level districts. Four middle schools were then selected from each selected district, resulting in a total of 128 schools stratified by school type and size. Next, two grade 7 classes and two grade 9 classes were randomly selected from each selected school. Finally, all students in the selected classes were included in the final sample. In subsequent follow-up waves, the 7th-grade cohort was followed continuously, while the 9th-grade cohort was not.

The current study used the first two waves of data on the 7th-grade cohort. In the baseline survey, a total of 10,275 7th-grade students were interviewed. One year later, during the 2014–2015 school year, 9449 of these students were successfully followed up. We excluded 808 participants, or 8.5 percent of the sample, due to missing values on variables of interest. Therefore, our final analytic sample consisted of 8641 students who were in grade 7 at wave 1 and in grade 8 at wave 2.

### 2.2. Measures

Outcomes. Both sleep duration and sleep problems recorded at the follow-up wave were examined. Sleep duration was assessed by self-report, using the following question: “On average, how much time do you sleep each night?” The variable was measured in hours and included in the analysis as a continuous variable. Sleep problems were also assessed by self-report, using the following question: “Do you suffer from the following sleeping problems under normal circumstances?”, with nine items of sleep problems, including “difficulty falling asleep”, “frequently waking up”, “drowsiness”, “inability to recover from fatigue after sleeping”, “snoring”, “teeth grinding”, “having dreams”, “sleep talking”, and “sleepwalking”. Following prior research [24], the total number of sleep problems, with a minimum of 0 and a maximum of 9, was included in the analysis.

Predictors. Three measures of time spent on private tutoring were examined. The CEPS collected data on time spent on private tutoring separately for weekdays and weekends at wave 2: “How much time on average everyday did you spend on private tutoring activities on weekdays/weekends?” Based on these two items, we additionally constructed an additional variable indicating the average hours of private tutoring per day during the week. In the following analysis, we would first pay attention to the “daily hours of tutoring during the week”, and then “daily hours of tutoring on weekdays” and “daily hours of tutoring on weekends”.

Control variables. The control variables were mainly related to respondents’ social demographic characteristics and educational background. Social demographic characteristics included age in years, gender (0 = male and 1 = female), ethnicity (0 = Han and 1 = ethnic minority), parental education, family economic condition, and sibling information (1 = only child and 0 = otherwise). Parental education was measured in years of schooling and was captured by the higher of the two parents’ educational attainment. Family economic condition, which was self-rated, had five values ranging from 1 (very poor) to 5 (very rich) and was treated as a continuous variable in the current study. The assessment of academic performance was quantified by calculating the mean of the last midterm exam scores in Chinese, mathematics, and English. The raw midterm scores were provided by the participating institutions. The data collectors used a standardization procedure to modify the raw scores to a mean of 70 and a standard deviation of 10 by school and grade because the scores may vary across schools in terms of the upper limit and may not be comparable between grades. Consequently, the average academic performance included in the analysis had a mean of 70. Table 1 presents descriptive statistics of the variables in our sample. Finally, we controlled for sleep duration at the baseline survey wave.

### 2.3. Analytical Strategy

We first performed a bivariate analysis, obtaining pairwise correlation coefficients between three tutoring variables and two sleep variables. We then turned to regression models to examine how each tutoring variable was related to each sleep variable while adjusting for other covariates.

The measures of sleep duration and number of sleep problems were continuous and count, respectively, so we used ordinary least square (OLS) regression for sleep duration and Poisson regression for number of sleep problems. We examined the effect of hours of tutoring per day regardless of whether the tutoring occurred on weekdays or weekends, and then examined the relative importance of hours of tutoring on weekdays and weekends. In all regression models, class dummies were also included to control for unobserved class-level characteristics or confounders shared by all individuals within the same classroom. Finally, to take advantage of the longitudinal nature of our data, we also controlled for sleep duration information collected at baseline as a lagged dependent variable. In this way, our results can be better interpreted in a causal framework.

## 3. Results

### 3.1. Descriptive Statistics

Table 1 summarizes descriptive statistics. Within our sample, the average age of respondents was 13.92 years (SD = 0.86). Approximately 49% of respondents identified as female, and approximately 8% identified as belonging to an ethnic minority. Parental education averaged 11.03 years (SD = 3.04). Family economic status, rated on a scale of 1 (lowest) to 5 (highest), had a mean score of 2.94 (SD = 0.61). Approximately 43% of the adolescents were only children. Participants’ academic performance had a mean score of 70.31 (SD = 8.75). Regarding sleep patterns, the adolescents reported an average of 8 h of sleep per night (SD = 1.10), with an average of 0.80 sleep problems experienced (SD = 1.13). Regarding time spent on private tutoring, the average time spent on tutoring per day was 0.76 h (SD = 1.28). When differentiating between weekdays and weekends, tutoring hours per day were 0.48 (SD = 1.05) on weekdays and 1.04 (SD = 1.89) on weekends, indicating that adolescents spent more time tutoring on weekends compared to weekdays.

### 3.2. Bivariate Analysis

Table 2 presents a correlation matrix showing the pairwise correlation coefficients between three tutoring variables and two sleep variables. First, the two sleep measures were negatively correlated (r = −0.105), indicating that longer sleep duration is associated with fewer sleep problems. Second, the three tutoring time measures were positively correlated, as expected. Third, each of the tutoring time measures was associated with each of the sleep measures, providing preliminary evidence that more time spent tutoring may lead to shorter sleep duration and greater sleep problems. All correlation coefficients reported in the table are statistically significant at least at the 0.05 level.

### 3.3. Regression Results

Table 3, Table 4 and Table 5 present the regression coefficients estimating the effects of tutoring time on sleep patterns, controlling for a number of covariates. Table 3 focuses on the average amount of time spent tutoring per day, regardless of whether the tutoring occurred on weekdays or weekends. The left two columns correspond to results from OLS regressions of sleep duration. Each additional hour of tutoring is associated with a significant decrease in average nighttime sleep of 0.017 h (*p* < 0.10), and the coefficient does not change much even after further controlling for respondents’ sleep information at the baseline wave (b = 0.016, *p* < 0.10). The right two columns correspond to the results of Poisson regressions of sleep problems. It is clear that more time spent on tutoring was associated with more sleep problems. Specifically, for every 1 h increase in tutoring time, the average number of reported sleep problems would increase by about 0.05 (*p* < 0.001). Taken together, the results suggest that daily hours of tutoring during the week have a negative association with hours of sleep and a positive association with the number of sleep problems.

Table 4 reports the results of OLS regressions of hours of sleep on weekday tutoring time and weekend tutoring time. In Model 1, only weekday tutoring time was included. In Model 2, only weekend tutoring time was included. Model 3 included both. In all models, weekend tutoring time had a negative effect on sleep duration, while weekday tutoring time did not play a significant role. Similarly, Table 5 presents the results of Poisson regressions of sleep problems on weekday and weekend tutoring time. Both weekday tutoring hours and weekend tutoring hours had a significant positive effect on sleep problems. For example, in Model 3 of Table 5, each 1 h increase in weekday tutoring was associated with a 0.037 increase in sleep problems (*p* < 0.01), and each 1 h increase in weekend tutoring was associated with a 0.018 increase in sleep problems. Note that the estimates were obtained after accounting for covariates and baseline sleep information.

## 4. Discussion

In recent decades, the amount and quality of sleep among adolescents have declined in many countries [6,7,8,9]. In social contexts where academic competition is intense, the role of academic activities in compromising adolescent sleep health has been much studied [10,11,12]. In this study, we paid particular attention to the association between time spent on private tutoring (also referred to as shadow education) and sleep patterns among Chinese adolescents. We used data from a nationwide two-wave panel survey of middle school students. We report results from OLS regressions of sleep hours at wave 2 and Poisson regressions of sleep problems at wave 2 on tutoring hours at wave 2, controlling for respondents’ demographic characteristics, educational background, and class-level differences. Our panel data also allowed us to introduce a lagged dependent variable, hours of sleep at wave 1, in all regression models to strengthen the causal interpretation of our findings. In addition to focusing on the “naïve” measure of tutoring hours, i.e., neglecting potential differences in tutoring hours between weekdays and weekends, we also examined the effects of tutoring hours on weekdays and weekends separately on sleep duration and sleep problems.

Would more hours of tutoring lead to shorter sleep duration and more sleep problems? This study provided largely confirmatory evidence. Ceteris paribus, an increase of 1 h per day of tutoring was associated with an average decrease of 0.016 h in nocturnal sleep duration and an average increase of 0.049 in sleep problems. Some previous studies have found similar results. For example, using the same data but a different research design than the current study, one study found that more time spent in private tutoring per week was associated with more sleep problems among Chinese adolescents [24]. More time spent on private tutoring was also found to be associated with shorter sleep duration among adolescents in South Korea [9] and Singapore [11]. To our knowledge, our study was among the first to examine the impact of tutoring time on both sleep duration and sleep problems. It should be noted that all existing evidence, including the results of the current study, is based on East Asian societies that share the same cultural tradition and emphasize academic competition and intensive parenting (e.g., helicopter parenting). In particular, the phenomenon of “helicopter parenting”, which can indeed be observed in many families, may play a role in shaping the associations between private tutoring and adolescent sleep patterns. This deserves scholarly attention in future research in East Asian contexts, as well as in other societies.

We also examined the independent roles of weekday and weekend tutoring in shaping sleep patterns. Our results consistently show that both weekday and weekend tutoring can have a positive impact on the number of sleep problems. In terms of sleep duration, weekend tutoring time played a negative role, while weekday tutoring time did not. It is likely that students’ weekday schedules were strictly regulated in terms of time to go to bed, time to go to school, and time to stay at school. Therefore, weekday sleep time cannot be easily displaced by tutoring practices. A recent study of Korean students found that more tutoring time was significantly associated with shorter sleep duration [25], which is different from our finding in this study. Unfortunately, the study did not examine the effect of weekend tutoring time. Further studies are warranted to reconcile the two contrasting findings and clarify possible mechanisms behind them.

The study has several limitations. First of all, we relied on self-reported measures of sleep duration and sleep problems, which can be subject to potential recall or reporting biases. Future research would benefit from employing more objective instruments to capture these variables, thereby enhancing the reliability and validity of the findings. Another limitation pertains to the sample employed in this research, which, despite being drawn from a national probability sample survey, exhibited a narrow grade range and included solely a cohort of grade 7 students at the baseline survey. In fact, private tutoring is also quite common at the elementary and senior high school levels [28]. Insofar as students in elementary school or senior high school may have distinct patterns of private tutoring participation and sleep patterns, our findings based on middle school students cannot be easily generalized to those populations. Future research focusing on students from a wide range of ages or grade levels may help to uncover the nuanced patterns regarding the relationship between tutoring and sleep health.

Despite its limitations, this study makes several contributions to our understanding of the relationship between private tutoring and adolescent sleep patterns. First, we used data from a national panel survey of middle school students in China, which may improve the generalizability and causality of our findings. Second, we focused on both sleep duration and sleep problems and associated them with both weekday and weekend tutoring hours, providing a more nuanced picture than previous studies. The results of the study may also be policy-relevant. Recently, the Chinese government implemented the so-called “double reduction policy”, which strictly prohibits after-school tutoring in order to promote the healthy development of students. Since tutoring has long been considered an important means of improving academic performance in Asian societies [18,19], the high demand for tutoring services was indeed difficult to reconcile [29,30]. In support of government policy, our findings can be used to better inform students and their parents in China that restricting private tutoring is beneficial for adolescents’ sleep quality. Finally, our study provides an important benchmark for future studies in other countries experiencing the expansion of private tutoring and the deterioration of adolescent sleep.

## Figures and Tables

**Table 1:** Descriptive statistics.

	Mean	SD	Min	Max
Hours of sleep	8.00	1.10	5	11
Number of sleep problems	0.80	1.13	0	8
Daily hours of tutoring during the week	0.76	1.28	0	6.5
Daily hours of tutoring on weekdays	0.48	1.05	0	4.5
Daily hours of tutoring on weekends	1.04	1.89	0	8.5
Age in years	13.92	0.86	12	17
Female	0.49	0.50	0	1
Ethnic minority	0.08	0.28	0	1
Parental education in years	11.03	3.04	0	19
Family economic condition	2.94	0.61	1	5
Only child	0.43	0.50	0	1
Academic performance	70.31	8.75	36.40	85.67
Hours of sleep: baseline	8.34	1.21	4	12

Descriptive statistics for all variables among 8641 adolescents in grade 8. For each variable, the mean, standard deviation (SD), minimum (Min), and maximum (Max) are reported. Data for all variables except the last one shown in the table were collected at the follow-up wave.

**Table 2:** Correlation matrix.

		(1)	(2)	(3)	(4)	(5)
(1)	Daily hours of tutoring during the week	1.000				
(2)	Daily hours of tutoring on weekdays	0.756	1.000			
(3)	Daily hours of tutoring on weekends	0.931	0.464	1.000		
(4)	Hours of sleep	−0.111	−0.052	−0.122	1.000	
(5)	Number of sleep problems	0.031	0.027	0.026	−0.105	1.000

Pairwise correlation coefficients between three tutoring variables and two sleep variables for 8641 adolescents in grade 8. All coefficients are statistically significant at least at the 0.05 level.

**Table 3:** Coefficients for daily hours of tutoring during the week in estimating hours of sleep and number of sleep problems.

	Hours of Sleep		Number of Sleep Problems	
	Model 1	Model 2	Model 3	Model 4
Daily hours of tutoring during the week	−0.017 *	−0.016 *	0.050 ***	0.049 ***
	[0.010]	[0.010]	[0.011]	[0.011]
Female	−0.141 ***	−0.125 ***	0.223 ***	0.217 ***
	[0.023]	[0.022]	[0.026]	[0.026]
Age (in years)	−0.029	−0.017	0.003	0.001
	[0.018]	[0.017]	[0.020]	[0.020]
Parental education in years	−0.002	0.001	0.007	0.006
	[0.005]	[0.005]	[0.005]	[0.005]
Family economic condition	0.045 *	0.027	−0.142 ***	−0.137 ***
	[0.021]	[0.020]	[0.023]	[0.023]
Only child	−0.008	0	−0.018	−0.021
	[0.027]	[0.026]	[0.030]	[0.030]
Ethnic minority	−0.034	−0.025	0.034	0.028
	[0.056]	[0.053]	[0.060]	[0.060]
Academic performance	−0.002	−0.001	−0.001	−0.002
	[0.002]	[0.002]	[0.002]	[0.002]
Hours of sleep: baseline		0.242 ***		−0.071 ***
		[0.009]		[0.011]
Class dummies	Controlled	Controlled	Controlled	Controlled
Constant	7.827 ***	5.707 ***	0.468	1.080 **
	[0.377]	[0.372]	[0.395]	[0.406]

Results from regressions of hours of sleep and number of sleep problems on daily hours of tutoring during the week for 8641 adolescents in grade 8. OLS regression models were used in estimating hours of sleep and Poisson regression models were used in estimating number of sleep problems. In brackets are standard errors. * *p* < 0.10, ** *p* < 0.01, *** *p* < 0.001.

**Table 4:** OLS regressions of sleep hours on daily hours of tutoring on weekdays and weekends.

	Model 1	Model 2	Model 3
Daily hours of tutoring on weekdays	−0.003		0.006
	[0.011]		[0.012]
Daily hours of tutoring on weekends		−0.014 *	−0.015 *
		[0.007]	[0.007]
Female	−0.125 ***	−0.124 ***	−0.124 ***
	[0.022]	[0.022]	[0.022]
Age in years	−0.017	−0.018	−0.018
	[0.017]	[0.017]	[0.017]
Parental education in years	0.001	0.002	0.002
	[0.005]	[0.005]	[0.005]
Family economic condition	0.025	0.027	0.026
	[0.020]	[0.020]	[0.020]
Only child	−0.001	0.001	0.001
	[0.026]	[0.026]	[0.026]
Ethnic minority	−0.024	−0.026	−0.026
	[0.053]	[0.053]	[0.053]
Academic performance	−0.001	−0.001	−0.001
	[0.002]	[0.002]	[0.002]
Hours of sleep: baseline	0.242 ***	0.242 ***	0.242 ***
	[0.009]	[0.009]	[0.009]
Class dummies	Controlled	Controlled	Controlled
Constant	5.701 ***	5.706 ***	5.704 ***
	[0.372]	[0.372]	[0.372]

Results from OLS regressions of hours of sleep on daily hours of tutoring on weekdays and on weekends for 8641 adolescents in grade 8. In brackets are standard errors. * *p* < 0.10, *** *p* < 0.001.

**Table 5:** Poisson regressions of sleep problems on daily hours of tutoring on weekdays and weekends.

	Model 1	Model 2	Model 3
Daily hours of tutoring on weekdays	0.049 ***		0.037 **
	[0.012]		[0.013]
Daily hours of tutoring on weekends		0.028 ***	0.018 *
		[0.007]	[0.008]
Female	0.218 ***	0.216 ***	0.217 ***
	[0.026]	[0.026]	[0.026]
Age in years	−0.001	0.000	−0.001
	[0.020]	[0.020]	[0.020]
Parental education in years	0.007	0.006	0.006
	[0.005]	[0.005]	[0.005]
Family economic condition	−0.135 ***	−0.136 ***	−0.137 ***
	[0.023]	[0.023]	[0.023]
Only child	−0.018	−0.02	−0.02
	[0.030]	[0.030]	[0.030]
Ethnic minority	0.024	0.029	0.027
	[0.060]	[0.060]	[0.060]
Academic performance	−0.001	−0.002	−0.001
	[0.002]	[0.002]	[0.002]
Hours of sleep: baseline	−0.072 ***	−0.071 ***	−0.071 ***
	[0.011]	[0.011]	[0.011]
Class dummies	Controlled	Controlled	Controlled
Constant	1.083 **	1.101 **	1.075 **
	[0.406]	[0.406]	[0.406]

Results from Poisson regressions of number of sleep problems on daily hours of tutoring on weekdays and on weekends for 8641 adolescents in grade 8. In brackets are standard errors. * *p* < 0.10, ** *p* < 0.01, *** *p* < 0.001.

## Data Availability

The data used in this study are available for application at http://ceps.ruc.edu.cn/, accessed on 18 February 2020.
